# Pampiniform Venous Plexus Thrombosis Presenting As Testicular Pain: Report of a Rare Case

**DOI:** 10.7759/cureus.95322

**Published:** 2025-10-24

**Authors:** Pronami Borah, Dharavath Venkatesh, Debarati Majumder, Saanjhi Rawat

**Affiliations:** 1 Radiodiagnosis, Assam Medical College, Dibrugarh, IND

**Keywords:** conservative management, doppler ultrasound, pampiniform plexus thrombosis, rare vascular cause, testicular pain

## Abstract

Spontaneous thrombosis of the pampiniform venous plexus is a rare vascular disorder that can manifest as acute testicular pain, often mimicking conditions such as epididymitis, orchitis, or testicular torsion. Due to its uncommon presentation, clinical diagnosis can be challenging, and a high level of suspicion is required. Scrotal Doppler ultrasonography serves as an effective, non-invasive diagnostic tool by demonstrating absent venous flow within the pampiniform plexus while confirming normal testicular perfusion. We present a case of spontaneous pampiniform venous plexus thrombosis diagnosed using Doppler ultrasound. The patient was successfully managed with conservative measures, including anticoagulation, analgesics, and brief bed rest, resulting in complete resolution of symptoms and normalization on follow-up imaging. This case underscores the importance of including pampiniform plexus thrombosis in the differential diagnosis of acute testicular pain and highlights the pivotal role of scrotal Doppler in guiding timely and appropriate treatment, thereby preventing unnecessary surgical intervention. Awareness of this rare entity is essential, particularly in outpatient and emergency settings, to ensure accurate diagnosis and optimal patient care.

## Introduction

Spontaneous thrombosis of the pampiniform venous plexus is extremely rare; fewer than 30 cases have been reported worldwide, making it a clinical curiosity that can easily be overlooked. Although it may occur in a wide age spectrum, its rarity and non-specific presentation often result in diagnostic uncertainty [1]. Patients typically present with acute scrotal pain, a clinical feature that overlaps with more common and urgent conditions such as testicular torsion, epididymo-orchitis, or incarcerated inguinal hernia [2,3]. Failure to recognize this entity may lead to unnecessary surgical exploration, which underscores the importance of timely and accurate diagnosis.

Scrotal Doppler ultrasonography remains the cornerstone of diagnosis. It enables real-time evaluation of pampiniform veins, revealing intraluminal echogenic thrombus, venous dilatation, absent or diminished venous flow, and preserved testicular arterial perfusion [4,5]. In certain cases, additional imaging modalities such as computed tomography (CT) or CT angiography may provide further anatomical details and help rule out other retroperitoneal or vascular pathologies. Nevertheless, Doppler ultrasound is favored in clinical practice due to its non-invasive nature, accessibility, and diagnostic reliability.

Management of pampiniform plexus thrombosis is generally conservative, consisting of analgesia, non-steroidal anti-inflammatory drugs, anticoagulation, and rest. Most patients show complete recovery without surgical intervention [6]. Given the diagnostic challenges and rarity of this condition, raising clinical awareness is essential, particularly in outpatient clinics and emergency departments. The present case highlights the diagnostic process, role of imaging, and successful conservative treatment of pampiniform plexus thrombosis, contributing to the existing body of literature and reinforcing its clinical relevance.

## Case presentation

A 43-year-old male with no prior significant medical history presented to the emergency department with a seven-day history of right testicular pain. The pain had a sudden onset and progressively increased in severity, and was associated with mild swelling and discomfort in the right inguinal region. The patient denied fever, lower urinary tract symptoms, urethral discharge, trauma, abdominal pain, vomiting, constipation, or chronic cough. He had no history of hematuria, flank pain, or renal swelling.

A comprehensive coagulation study (prothrombin time (PT), activated partial thromboplastin time (aPTT), and international normalized ratio (INR)) was performed, with all results found to be within normal limits.

**Figure 1:**
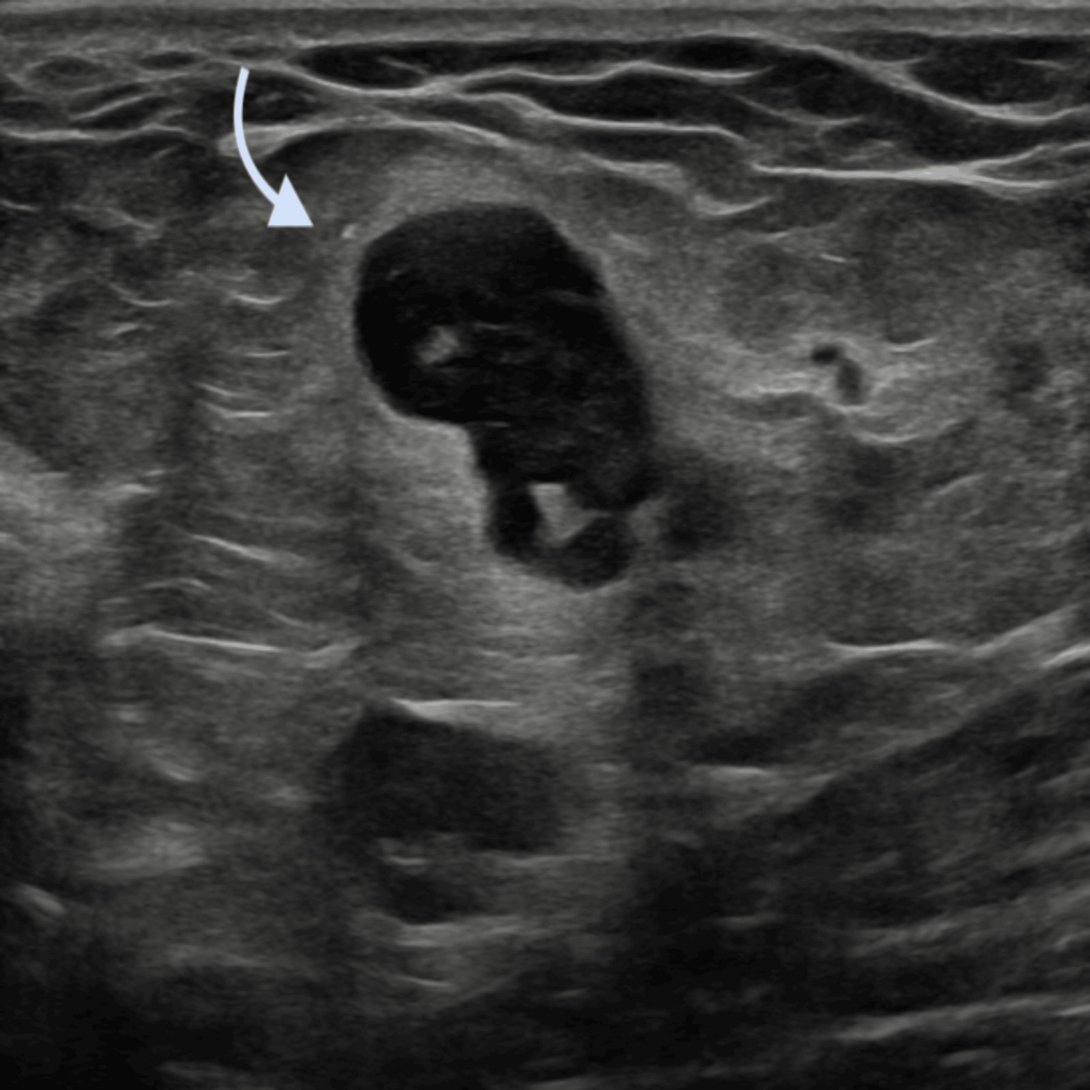
Enlarged inguinal lymph nodes

On physical examination, mild tenderness and induration were observed in the right inguinal region, which was consistent with enlarged inguinal lymph nodes (Figure 1). The right testis was tender but otherwise normal in size, shape, and consistency. Abdominal examination was unremarkable, with no palpable masses or flank tenderness.

**Figure 2:**
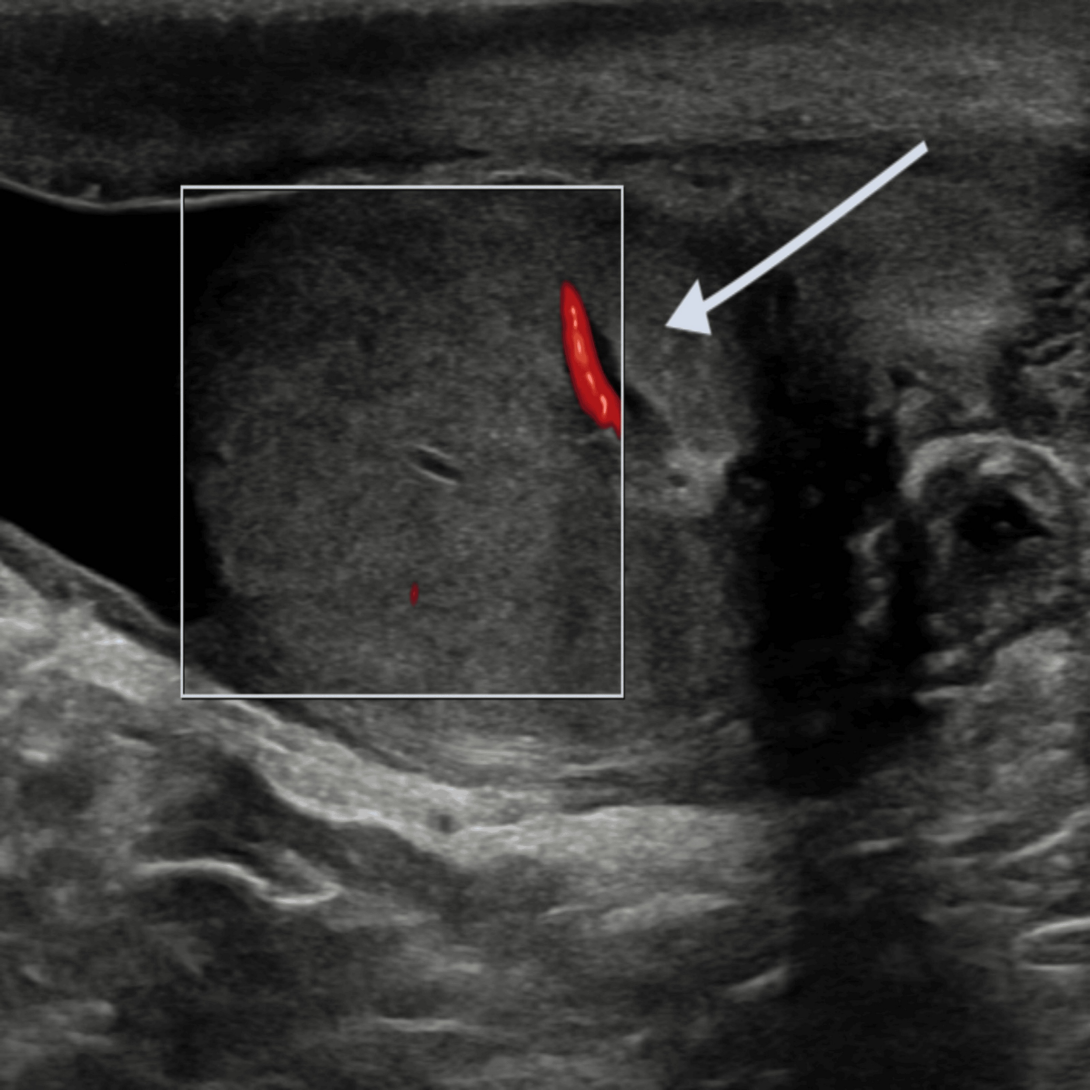
Color Doppler image demonstrating preserved testicular perfusion.

Scrotal Doppler ultrasonography demonstrated a dilated vein within the right spermatic cord containing echogenic intraluminal material, findings consistent with thrombosis of the pampiniform venous plexus; testicular perfusion was preserved (Figure 2).

**Figure 3:**
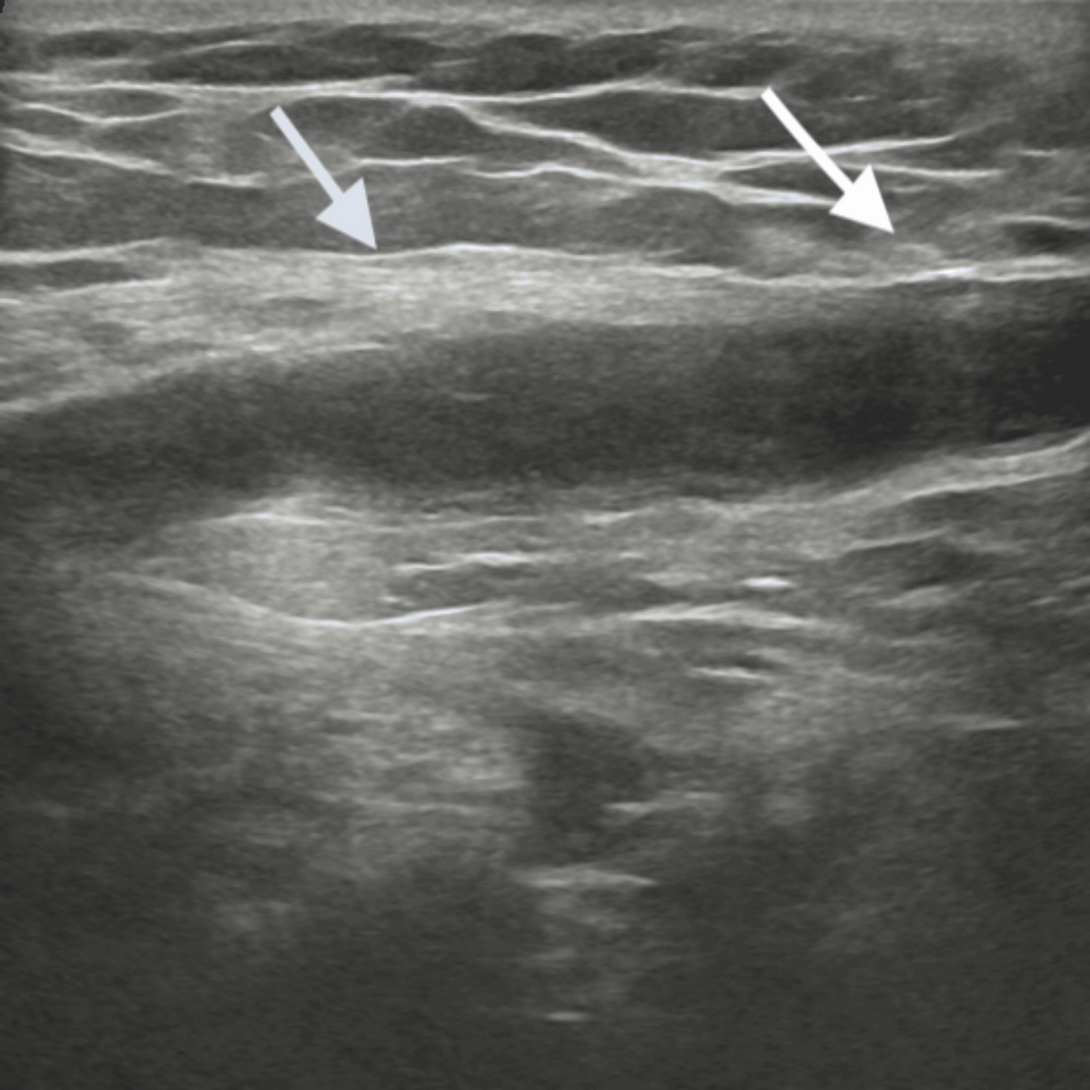
Thrombosed vein within the pampiniform plexus in the spermatic cord.

Ultrasound confirmed an enlarged pampiniform venous channel containing echogenic material consistent with thrombus (Figure 3).

**Figure 4:**
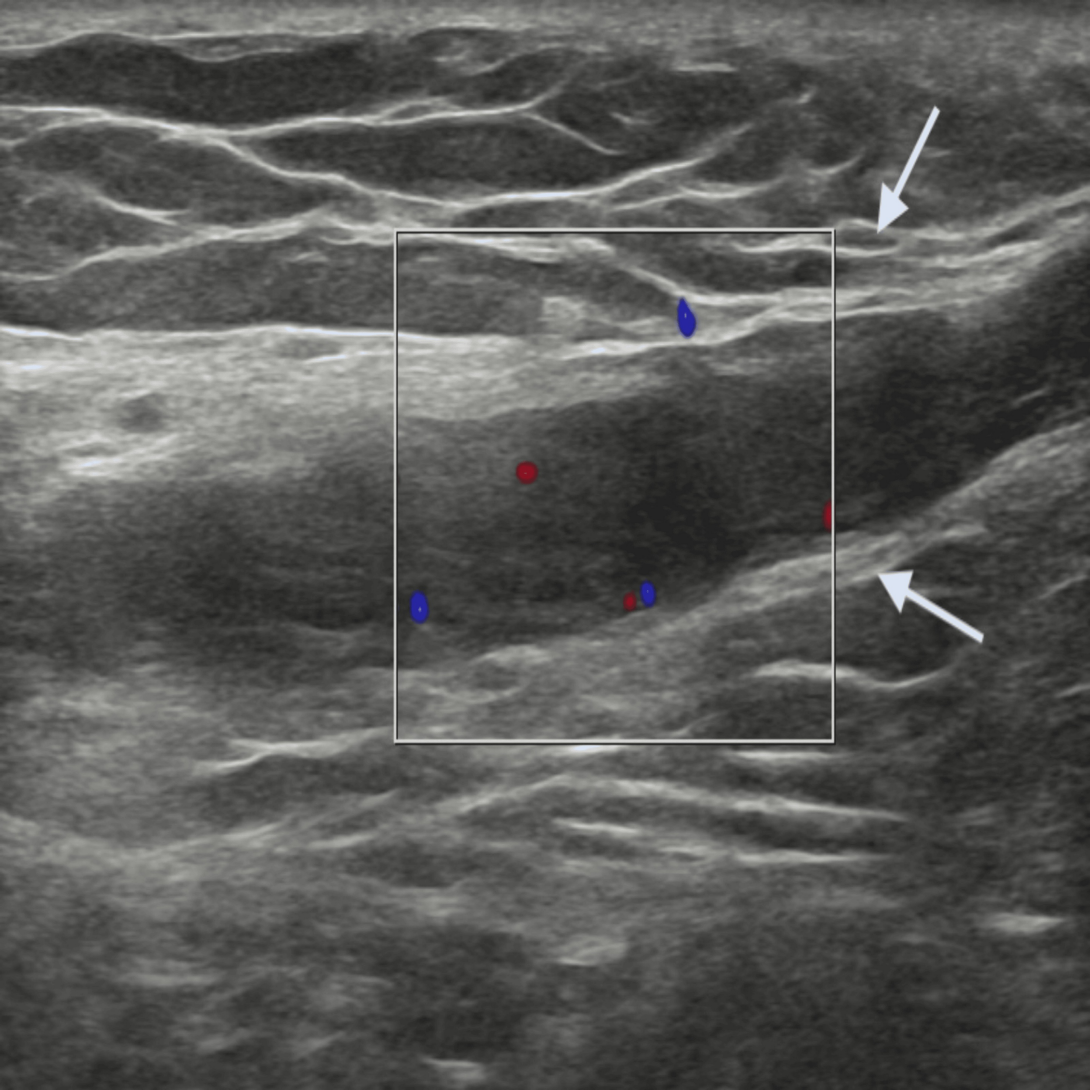
Color Doppler ultrasound of the right pampiniform venous plexus demonstrating absence of vascular flow within the thrombosed vein

Color Doppler of the pampiniform venous plexus demonstrated absence of vascular flow within the thrombosed vein on the right side (Figure 4).

The patient was admitted and managed conservatively with bed rest, anti-inflammatory medications, analgesics, and a therapeutic course of anticoagulation. Symptomatic improvement was observed over 7-10 days. He was discharged in stable condition. Follow-up Doppler ultrasonography performed two months later showed complete resolution of the thrombus.

Table 1 presents the clinical timeline of the case from symptom onset to resolution.

**Table 1:** Clinical timeline of the case: symptom onset → imaging → treatment → resolution PT: prothrombin time; aPTT: activated partial thromboplastin time; INR: international normalized ratio; CBC: complete blood count; NSAID: non-steroidal anti-inflammatory drug

Event	Timing	Details
Symptom onset	Day 0	Sudden right testicular pain with mild swelling; no trauma or systemic symptoms
Initial evaluation & labs	Day 7 (on admission)	Comprehensive coagulation profile: PT, aPTT, INR—all normal; CBC and metabolic panel normal; no history of coagulopathies or systemic risk factors
Imaging (Doppler USG)	Day 7	Dilated pampiniform vein, echogenic thrombus; absent venous flow; preserved arterial perfusion
Treatment initiated	Day 7	Bed rest, analgesics, NSAIDs, anticoagulation
Resolution (follow-up)	2 months after onset	Complete clinical and sonographic resolution of thrombosis

## Discussion

Pampiniform venous plexus thrombosis represents a rare cause of acute scrotal pain and is frequently underrecognized due to its clinical similarity with other scrotal pathologies [2,3]. Misdiagnoses have been reported, with patients initially suspected of having testicular torsion or incarcerated inguinal hernia, sometimes even undergoing surgical exploration before the correct diagnosis was established [4]. This reinforces the need for heightened clinical suspicion when evaluating patients with acute scrotal pain.

The underlying etiology of spontaneous thrombosis remains uncertain. Proposed mechanisms include venous stasis, hypercoagulable states, dehydration, local trauma, strenuous physical exertion, or increased venous pressure following vigorous sexual activity [5,6]. Associations with systemic diseases such as Buerger’s disease and other vasculopathies have also been postulated, though definitive causality remains unclear [7]. While most cases occur on the left side, likely due to anatomical factors such as the longer left testicular vein draining into the left renal vein at a right angle, right-sided cases-such as the one reported here-demonstrate that laterality should not exclude consideration of this diagnosis [8].

Doppler ultrasonography is both diagnostic and reassuring in this setting. The hallmark features include non-compressible, dilated pampiniform veins containing echogenic material consistent with thrombus and absence of venous flow, while intratesticular perfusion is preserved [4,5]. This distinction from testicular torsion, where arterial inflow is compromised, is crucial. In rare circumstances where ultrasound findings are equivocal, CT or MR venography may be warranted to confirm the diagnosis or exclude retroperitoneal and renal venous pathologies [1,9].

Treatment strategies are largely conservative. Most reports describe successful outcomes with a combination of analgesics, non-steroidal anti-inflammatory drugs, anticoagulation, and rest, leading to symptom resolution and radiological improvement [6,10]. Our patient demonstrated significant improvement within days and achieved complete resolution on follow-up imaging, consistent with prior observations. Surgical intervention is rarely required, though it may be considered in cases of diagnostic uncertainty, severe refractory pain, or complications [4]. Importantly, correct preoperative identification of this entity prevents unnecessary orchiectomy or varicocelectomy.

The clinical implications of this case extend to both emergency and outpatient settings, where physicians frequently encounter acute scrotal pain. Early consideration of pampiniform plexus thrombosis in the differential diagnosis may reduce the risk of mismanagement. Furthermore, the absence of standardized diagnostic and treatment guidelines reflects the need for larger case series and multicenter analyses. Future research should aim to define risk factors, establish diagnostic algorithms, and evaluate long-term outcomes to better inform clinical practice.

## Conclusions

Spontaneous thrombosis of the pampiniform venous plexus is an exceedingly rare but clinically significant cause of acute scrotal pain. Its presentation closely mimics other urgent conditions such as testicular torsion and epididymo-orchitis, which often leads to misdiagnosis. Scrotal Doppler ultrasonography remains the diagnostic modality of choice, allowing differentiation from surgical emergencies by confirming venous thrombosis while preserving testicular perfusion. Conservative management with analgesics, anti-inflammatory agents, and anticoagulation is usually sufficient and highly effective. Increased awareness of this entity among clinicians, particularly in emergency and outpatient settings, can prevent unnecessary surgical exploration and optimize patient outcomes.
